# Striatal increase of dopamine receptor 2 density in idiopathic and syndromic mouse models of autism spectrum disorder

**DOI:** 10.3389/fpsyt.2023.1110525

**Published:** 2023-03-08

**Authors:** Stuti Chhabra, Leonardo Nardi, Petra Leukel, Clemens J. Sommer, Michael J. Schmeisser

**Affiliations:** ^1^Institute of Anatomy, University Medical Center of the Johannes-Gutenberg University, Mainz, Germany; ^2^Focus Program Translational Neurosciences, University Medical Center of the Johannes Gutenberg-University, Mainz, Germany; ^3^Institute of Neuropathology, University Medical Center of the Johannes-Gutenberg University, Mainz, Germany

**Keywords:** ASD, autism (ASD), dopamine receptor (d1r and d2r), dorsal striatum, ventral striatum, receptor autoradiography

## Abstract

Autism spectrum disorder (ASD) comprises a wide range of neurodevelopmental phenotypes united by impaired social interaction and repetitive behavior. Environmental and genetic factors are associated with the pathogenesis of ASD, while other cases are classified as idiopathic. The dopaminergic system has a profound impact in the modulation of motor and reward-motivated behaviors, and defects in dopaminergic circuits are implicated in ASD. In our study, we compare three well-established mouse models of ASD, one idiopathic, the BTBR strain, and two syndromic, *Fmr1* and *Shank3* mutants. In these models, and in humans with ASD, alterations in dopaminergic metabolism and neurotransmission were highlighted. Still, accurate knowledge about the distribution of dopamine receptor densities in the basal ganglia is lacking. Using receptor autoradiography, we describe the neuroanatomical distribution of D1 and D2 receptors in dorsal and ventral striatum at late infancy and adulthood in the above-mentioned models. We show that D1 receptor binding density is different among the models irrespective of the region. A significant convergence in increased D2 receptor binding density in the ventral striatum at adulthood becomes apparent in BTBR and *Shank3* lines, and a similar trend was observed in the *Fmr1* line. Altogether, our results confirm the involvement of the dopaminergic system, showing defined alterations in dopamine receptor binding density in three well-established ASD lines, which may provide a plausible explanation to some of the prevalent traits of ASD. Moreover, our study provides a neuroanatomical framework to explain the utilization of D2-acting drugs such as Risperidone and Aripiprazole in ASD.

## Introduction

Autism spectrum disorder (ASD) is a range of neurodevelopmental conditions defined by impairment in social interaction and repetitive behavior. These core symptoms are accompanied by other comorbidities such as cognitive disabilities, anxiety, sleep disturbances, hyperactivity, and motor impairments. The broad span of ASD symptoms can affect each individual to a varying degree of severity (1). Global prevalence of ASD has risen in recent years, with 1 in 100 children being affected across all socioeconomic, racial, and ethnic groups (2). However, etiology of ASD is still poorly understood, with no single underlying cause, rather a complex interplay between genetic and environmental factors that alone or in combination are implicated in development of the disorder (1, 3). Heterogeneous presentation of the disease reveals that different brain regions and discrete perturbances in specific neural circuits contribute to the distinct features of ASD symptomatology (4).

The dopaminergic system has been characterized in controlling motor functions and reward-motivated behavior. Dopaminergic neurons are found mainly in the substantia nigra, pars compacta, and in the ventral tegmental area (VTA). The projections arising in the substantia nigra, pars compacta target mainly the dorsal striatum (DS), forming the nigrostriatal pathway, which is relevant in the control of voluntary movement (5). The axons from neurons, which reside in the VTA, project mainly to the Ventral striatum (VS) and prefrontal cortex, making up the mesocorticolimbic pathway, involved in reward, motivation and emotion (6). Dopamine (DA) receptors belong to the family of G protein–coupled receptors. According to pharmacological and binding studies, DA receptors are grouped into D1-like (D1 and D5) and D2-like (D2, D3, and D4) families, depending upon their ability to regulate positively or negatively intracellular concentration of cyclic adenosine monophosphate, respectively (7).

In recent years, a possible role of a dysfunctional dopaminergic system in ASD has gained a lot of attention. Multiple studies have highlighted the association between alterations in the dopaminergic system and ASD (8–10). However, the impact of these changes in contributing to ASD pathophysiology still needs to be further investigated. Additional evidence in support of this view comes from the fact that the only FDA approved drugs for ASD are the antipsychotics risperidone and aripiprazole, which act as D2 receptor antagonist and partial agonist, respectively (11, 12). Neuroanatomical alterations of the basal ganglia have been discovered in individuals with ASD. Significant changes in volume and neuronal density were reported in the DS (13). Also, a significantly smaller bilateral VS and larger cerebral ventricles were identified in individuals with a later diagnosis of ASD (14). In mice, it has also been observed that chemogenetic inhibition of dopaminergic neurons in the VTA leads to reduced exploration of novel social stimuli (15). As a result of the abundant evidence available, a DA hypothesis of ASD has been advanced. It has been postulated that variations in the nigrostriatal pathway may lead to motor impairments and stereotyped behaviors in subjects with ASD. Also, any alterations in the mesocorticolimbic pathway could lead to social deficits, reduced motivation, and changes in reward-related behavior (16–18).

Various mouse models recapitulating ASD-like phenotypes are widely utilized as preclinical tools to understand the causal role of genetic and environmental factors. Approximately 75% of ASD is idiopathic. Remaining cases show a specific genetic cause, such as fragile X syndrome, tuberous sclerosis and Phelan-McDermid syndrome (19). For this study, we chose three well-established mouse strains that recapitulate ASD features. Black and tan brachyury (BTBR) mice are an inbred strain characterized by the lack of corpus callosum and a severe reduction of hippocampal commissure (20). This line provides face validity for the majority of core symptoms of ASD, making it a suitable model for idiopathic ASD (21). Fragile X mental retardation 1 (*Fmr1*) knockout mice are the most investigated model for the human fragile X syndrome, the most common cause for intellectual disability and ASD in human. This gene codes for a mRNA binding protein (FMRP), whose activity is essential for proper synaptic plasticity and architecture (22). SH3 and multiple ankyrin repeat domain 3 (SHANK3) is a postsynaptic scaffold protein, which interacts with several postsynaptic receptors, signaling molecules, and cytoskeletal proteins (23). Mutations of SHANK3 are associated with the Phelan-McDermid syndrome and ASD (24). It has been inferred from previous studies that these three mouse lines display altered D1 and D2 receptor mediated neurotransmission and striatal pathway disruption, thus making the selected mouse strains a suitable choice for this study (25–28).

In an attempt to unravel the pathomechanistic underpinnings of a dysfunctional dopaminergic system in ASD, we investigated the alteration of DA receptor densities in the three above-mentioned ASD mouse lines in an age and region-dependent fashion.

## Materials and methods

### Animals

BTBR (BTBR *T^+^ Itpr^tf^*/J, stock #002282), C57BL6/J (stock #000664), *Fmr1* (B6.129P2-*Fmr1^tm1Cgr^*/J, stock #003025), and *Shank3b* (B6.129-*Shank3^tm2Gfng^*/J, stock #017688) mice were purchased from Jackson laboratories and housed in a pathogen-free facility with 12 h light/dark cycle, food, and water available *ad libitum. Fmr1^−/y^* (*Fmr1* KO) mice were generated by (29). *Shank3b* (B6.129-*Shank3^tm2Gfng^*/J) mice were generated by replacing exons 13–16 with a neomycin resistance cassette (26). BTBR, *Fmr1^−/y^* (*Fmr1* KO), and *Shank3b^−/−^* (*Shank3b* KO) were used as test animals. C57BL6/J mice were used as controls for BTBR mice, wildtype littermates for *Fmr1* KO and *Shank3b* KO. Breeding was approved by the local authorities. Only male mice were used in the experiments, seen the higher prevalence of ASD in male individuals (2). The number of animals tested in each experiment is reported in every figure legend. All the experiments were performed according to guidelines of the central animal facility institution (TARC, Mainz University Medical Center) representing those of the German Animal Welfare Act and the European Directive 2010/63/EU for the protection of animals used for scientific purposes. Reporting was carried out according to the ARRIVE guidelines for reporting *in vivo* experiments.

### Tissue collection and processing

After decapitation, brains were rapidly removed, frozen in isopentane and stored at −80°C until cutting. The brains were serially cut (20 μm thickness) in the coronal plane with a cryostat microtome (Leica, Germany). Totally, 4 slices were collected between the bregma points 1.53 mm 0.97 mm according to Paxinos and Franklin (30), where both DS and VS can be properly visualized. The first two slices were used for histological staining, the successive two were incubated with the [H^3^]-labeled ligands for D1 and D2 receptors. The slices were stored at −80°C until further histological and autoradiographic experiments.

### Histology

Hematoxylin–eosin staining was performed to help spatially localize the DS and VS on the autoradiograms. Briefly, the frozen brain slices were acclimatized at room temperature for 10 min. The slices were then incubated in acetone for 5 min, briefly air dried, and dipped in hematoxylin (Thermo Fisher) for 1 min. After washing in running water for 10 min, the slices were put for 10 s in Eosin Y (Thermo Fisher). Then, the slices were dehydrated in increasing ethanol concentrations (96 and 100%) each for 2 min. Finally, the slices were placed for 3 min in xylol, and cover slipped with Cytoseal XYL (Thermo Fisher). Pictures were scanned at 4× magnification with a Leica microscope (Leica, Germany), digitized, and transferred to the MCID program.

### *In vitro* receptor autoradiography

The receptor binding density for D1 and D2 receptors was adapted from the protocols described in Sommer et al. (31) and Behuet et al.’s study (32). The tritiated ligands [^3^H]-SCH23390 and [^3^H]-Raclopride were purchased from PerkinElmer (Germany). SCH23390 is a potent D1 and D5 receptor antagonist. Raclopride is a D2 and D3 selective receptor antagonist. Both ligands bind to the receptors localized on the cell surface, without penetrating inside the cell membrane. In the first step, the pre-incubation, endogenous ligands were washed off. In the following main incubation, the tritiated ligands were incubated both in the presence of a competitor, in order to determine the unspecific binding, and without it, in order to assess the total binding. Finally, the slices were rinsed. The slices incubated with [^3^H]-Raclopride were additionally dried with a cold air stream. A detailed description of the protocols used is reported in Table 1.

**Table 1:** Receptor binding protocols for the dopaminergic [^3^H] ligands with competitors (noted with *) and incubation conditions.

Receptor-[^3^H] ligand	Procedure	Incubation buffer	Time/temperature
	Pre-incubation	50 mM Tris–HCl (pH 7.4)	
	Main incubation	50 mM Tris–HCl (pH 7.4)	
	Rinsing	50 mM Tris–HCl (pH 7.4)	
Dopamine 1-[^3^H]SCH23390	Pre-incubation	+120 mM NaCl	20 min at 22°C
		+5 mM KCl	
		+2 mM CaCl_2_	
		+1 mM MgCl_2_	
	Main incubation	+120 mM NaCl	90 min at 22°C
		+5 mM KCl	
		+2 mM CaCl_2_	
		+1 mM MgCl_2_	
		+1 μM Mianserin	
		+1.67 nM [^3^H]SCH23390	
		+1 μM SKF 83566*	
	Rinsing	+120 mM NaCl	2 × 10 s at 4°C
		+5 mM KCl	
		+2 mM CaCl_2_	
		+1 mM MgCl_2_	
Dopamine 2-[^3^H]Raclopride	Pre-incubation	+0.1% Ascorbate	20 min at 22°C
		+150 mM NaCl	
	Main incubation	+0.1% Ascorbate	45 min at 22°C
		+150 mM NaCl	
		+0.3 nM [^3^H]Raclopride	
		+1 μM Butaclamol*	
	Rinsing	+0.1% Ascorbate	6 × 1 min at 4°C
		+150 mM NaCl	

### Image acquisition and analysis

**Figure 1:**
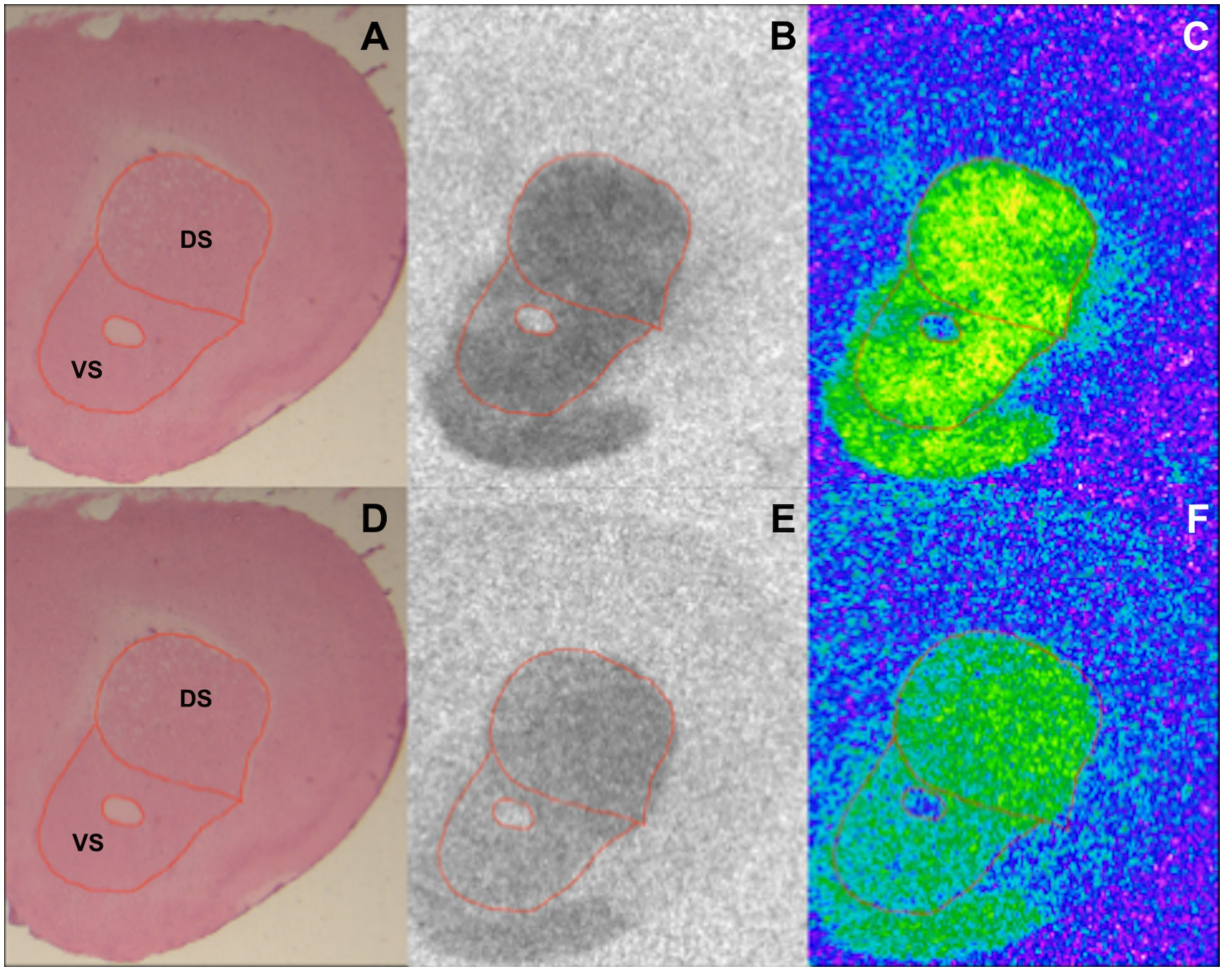
Exemplary section from a BTBR 12-week-old mouse showing DS and VS in an HE stained section **(A,D)**, autoradiographic image of binding to D1 **(B)** and D2 receptors **(E)**, and the corresponding color-coded picture of the same autoradiogram [**(C,F)** respectively].

Image acquisition and analysis were performed as described in Mammele et al.’s study (33). [^3^H] plastic standards (Microscales^®^; Amersham, Freiburg, Germany) were exposed together with the tritium-labeled sections to a [^3^H]-sensitive film (Bio Max MR-1 Autoradiography Film, KODAKTM) for 7 ([^3^H]-SCH23390) and 10 weeks ([^3^H]-Raclopride). The autoradiograms and the standards were scanned in equal lighting conditions with the digital CoolSNAP camera (Roper Scientific, Photometrics CoolSNAPTM *cf.*, Ottobrunn/Munich Germany) and digitized with the MCID image analysis system (Imaging Research Inc., St. Catharines, Ontario, Canada). The standards were used to calculate the relationship between the gray values of the autoradiograms and the concentration of radioactivity. Total binding was calculated on the autoradiograms on both hemispheres in DS and VS after tracing the boundary of the region of interest on the hematoxylin–eosin staining (Figure 1). The unspecific binding was consistently slightly above background signal or completely lacking. The value was then subtracted from the total binding.

### Statistical analysis

Statistical analysis was carried out with Prism (GraphPad, Version 9). Student’s *t*-test was performed. *p* < 0.05 was taken as threshold for statistical significance and results are shown as the mean ± SEM. The autoradiography experiments were performed in a blinded manner. Data were expressed as percentage of controls.

## Results

HE-stained sections were used to trace DS and VS (Figures 1A,D). The boundaries of the regions of interest were then overlapped on the autoradiograms for D1 and D2 receptors prior to analysis (Figures 1B,C,E,F, respectively). The analysis of the binding density to D1 and D2 receptors revealed various alterations in DS and VS of both 4- and 12-week-old BTBR, *Fmr1* KO, and *Shank3b* KO mice.

**Figure 2:**
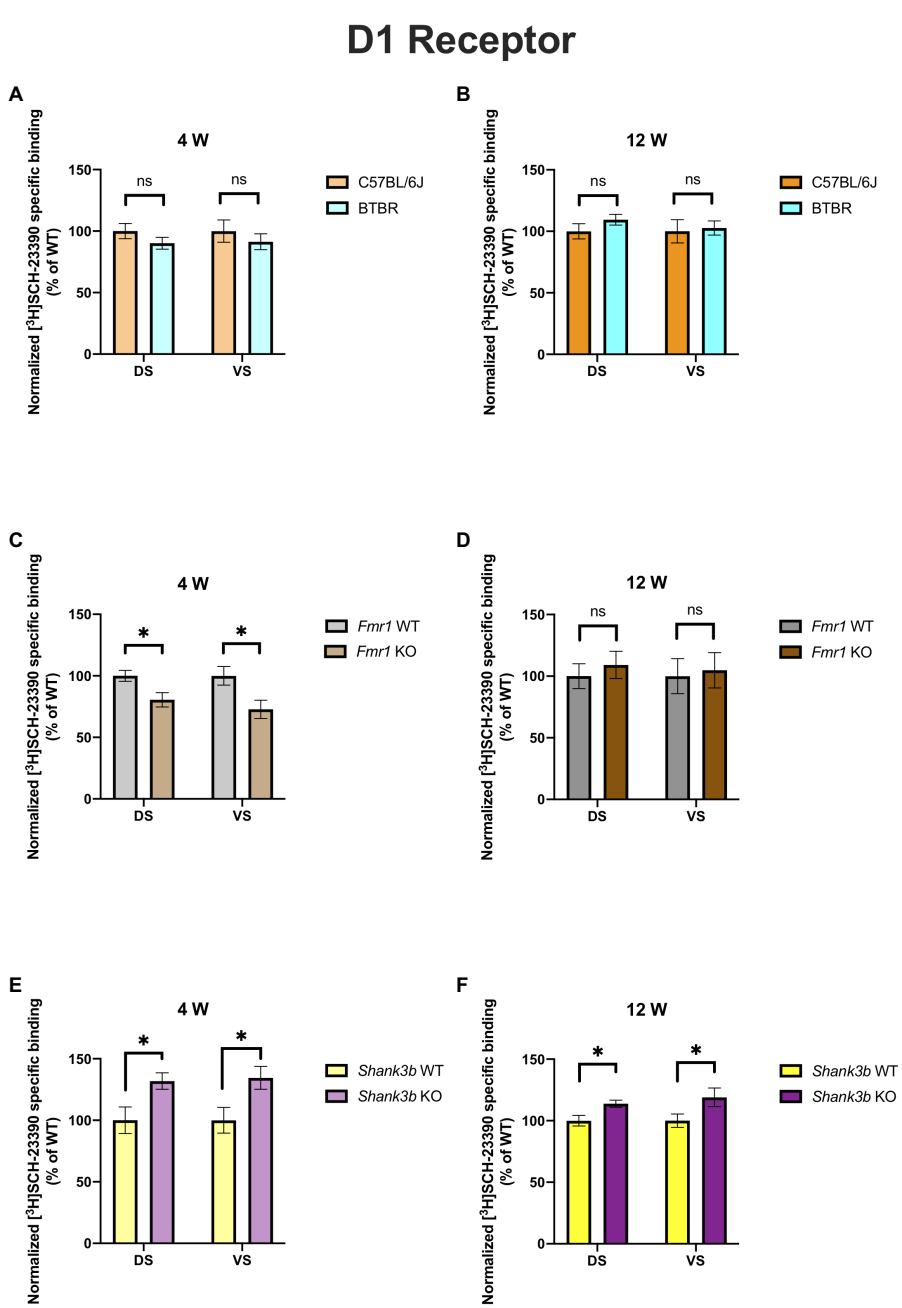
Bar charts representing mean and SEM of the receptor density for D1 receptors. DS: C57BL6/J (*n* = 8), BTBR (*n* = 8) and *VS*: C57BL6/J (*n* = 8), BTBR (*n* = 8) at 4 weeks of age **(A)**; DS: C57BL6/J (*n* = 10), BTBR (*n* = 11) and *VS*: C57BL6/J (*n* = 10), BTBR (*n* = 11) at 12 weeks of age **(B)**; DS: *Fmr1* WT (*n* = 9), *Fmr1* KO mice (*n* = 8) and *VS*: *Fmr1* WT (*n* = 9) and *Fmr1* KO mice (*n* = 7) at 4 weeks of age **(C)**; DS: *Fmr1* WT (*n* = 7), *Fmr1* KO mice (*n* = 8) and *VS*: *Fmr1* WT (*n* = 7) and *Fmr1* KO mice (*n* = 8) at 12 weeks of age **(D)**; DS: Shank*3b* WT (*n* = 5), *Shank3b* KO mice (*n* = 6) and *VS*: Shank*3b* WT (*n* = 5), *Shank3b* KO mice (*n* = 6) at 4 weeks of age **(E)**; DS: Shank*3b* WT (*n* = 14), *Shank3b* KO mice (*n* = 12) and *VS*: Shank*3b* WT (*n* = 14) and *Shank3b* KO mice (*n* = 12) at 12 weeks of age **(F)**. Significant differences are indicated with an asterisk (*p < 0.05). Changes are represented as percentage of the mean of C57BL6/J, *Fmr1* WT, *Shank3b* WT mice, respectively.

**Figure 3:**
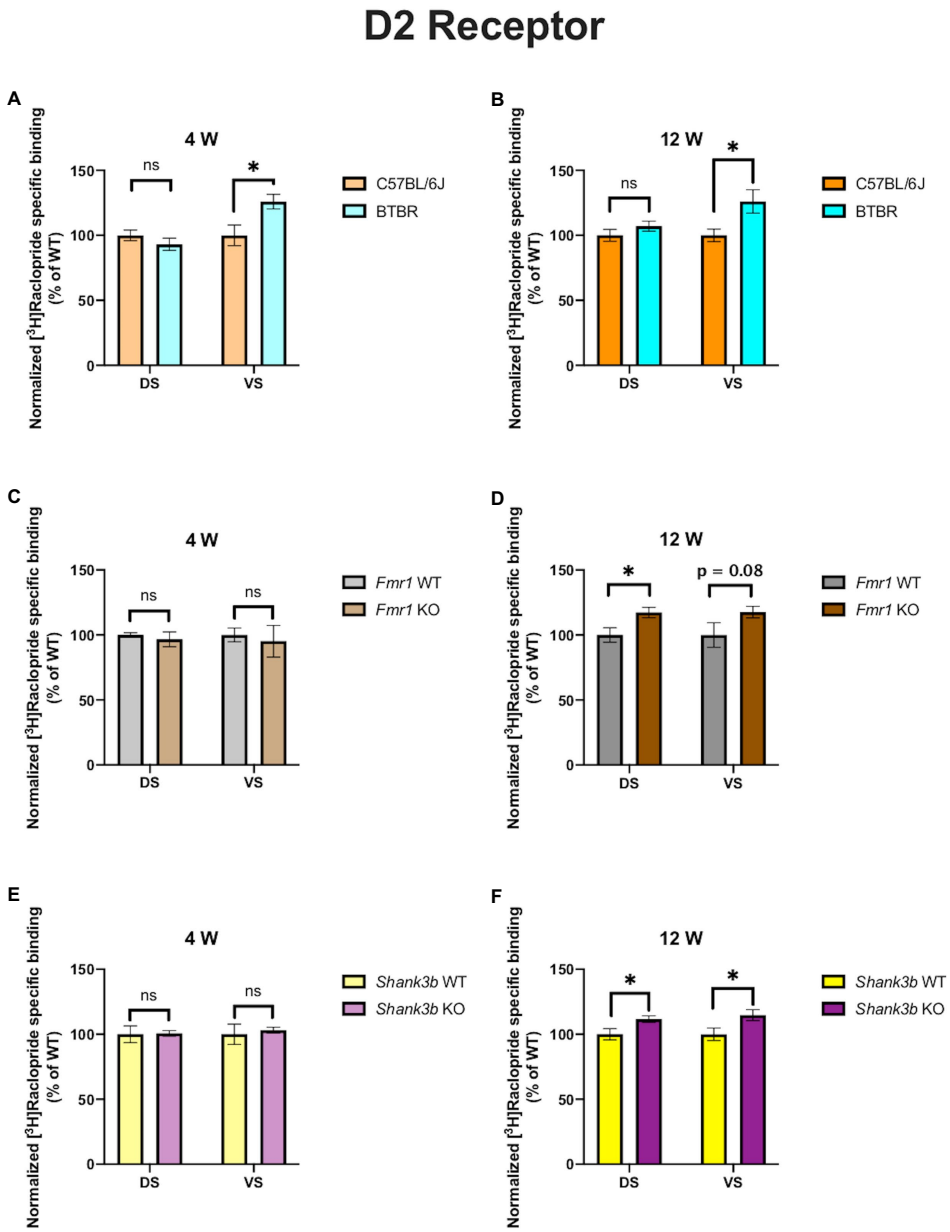
Bar charts representing mean and SEM of the receptor density for D2 receptors. DS: C57BL6/J (*n* = 9), BTBR (*n* = 8) and *VS*: C57BL6/J (*n* = 9), BTBR (*n* = 6) at 4 weeks of age **(A)**; DS: C57BL6/J (*n* = 10), BTBR (*n* = 9) and *VS*: C57BL6/J (*n* = 10), BTBR (*n* = 7) at 12 weeks of age **(B)**; DS: *Fmr1* WT (*n* = 8), *Fmr1* KO mice (*n* = 8) and *VS*: *Fmr1* WT (*n* = 8) and *Fmr1* KO mice (*n* = 8) at 4 weeks of age **(C)**; DS: *Fmr1* WT (*n* = 5), *Fmr1* KO mice (*n* = 8) and *VS*: *Fmr1* WT (*n* = 5) and *Fmr1* KO mice (*n* = 8) at 12 weeks of age **(D)**; DS: Shank*3b* WT (*n* = 6), *Shank3b* KO mice (*n* = 6) and *VS*: Shank*3b* WT (*n* = 6), *Shank3b* KO mice (*n* = 6) at 4 weeks of age **(E)**; DS: Shank*3b* WT (*n* = 12), *Shank3b* KO mice (*n* = 12) and *VS*: Shank*3b* WT (*n* = 12) and *Shank3b* KO mice (*n* = 11) at 12 weeks of age **(F)**. Significant differences are indicated with an asterisk (*p < 0.05). Changes are represented as percentage of the mean of C57BL6/J, *Fmr1* WT, *Shank3b* WT mice, respectively.

In 4-week-old BTBR mice, D1 receptor binding density was unaltered in both DS (*p* = 0.23) and VS (*p* = 0.45) (Figure 2A). At the same time point, no difference could be found in D2 receptor binding density in DS (*p* = 0.28), whereas it was increased in VS (*p* = 0.03) (Figure 3A). A similar pattern was observed also in 12-week-old BTBR mice. Binding density to D1 receptors was not changed both in DS (*p* = 0.22) and in VS (*p* = 0.8) (Figure 2B); D2 binding density remained unchanged in DS (*p* = 0.25) but was increased in VS (*p* = 0.014) (Figure 3B). Hence, VS showed at both time points increased D2 receptor binding density, while binding density to D1 receptors was not altered at either time point.

*Fmr1* KO mice showed a reduced receptor binding density to D1 receptors both in DS (*p* = 0.01) and in VS (*p* = 0.04) (Figure 2C) but no changes for D2 receptor binding density (DS: *p* = 0.58; *VS p* = 0.72) at 4 weeks (Figure 3C). At 12 weeks no alteration was found in the binding density to D1 receptors (DS: *p* = 0.56; VS: *p* = 0.82) (Figure 2D). The binding profile to D2 receptors was instead significantly increased in DS (*p* = 0.02) and showed a strong tendency in the same direction also in VS (*p* = 0.08) (Figure 3D). For *Fmr1* KO mice, it is hence possible to observe both in DS and VS at 4 weeks reduction of D1 binding density. Interestingly, this change was not observed at 12 weeks, implicating a possible role of developmental compensatory changes. Interestingly, the D2 binding density had an opposite dynamic in DS, being unmodified at 4 weeks, but increased at 12 weeks. The changes described at 12 weeks hence mirrors what we observed in the BTBR line.

*Shank3b* KO showed an increase of D1 receptor binding density in both DS and VS at 4 (DS: *p* = 0.02; VS: *p* = 0.03) (Figure 2E) and 12 weeks (DS: *p* = 0.01; VS: *p* = 0.04) (Figure 2F). With respect to D2 receptors, no change was found at 4 weeks (DS: *p* = 0.92; *VS*: *p* = 0.7) (Figure 3E), whereas an increased binding density was registered at 12 weeks in both regions (DS: *p* = 0.02; VS: *p* = 0.03) (Figure 3F). Taken together, we show that diverse ASD mouse models have a convergent increase in the D2 binding density in the VS at adulthood, implicating that the mesolimbic circuitry might be a common denominator in ASD pathophysiology. This brain region could therefore be acknowledged as a point of commonality among various ASD models in the context of dopamine signaling, thus making it an interesting candidate for further exploration. We also observed increased D2 binding in the DS of *Fmr1* KO and *Shank3b* KO at adulthood. D1 binding density, on the contrary, showed no convergent changes among mouse lines at both time points, but an individual phenotypic profile.

## Discussion

To our knowledge, we provide the first thorough neuroanatomical characterization of D1 and D2 receptor distribution by the means of receptor autoradiography in the DS and VS of several ASD mouse models at different developmental stages. For our analysis, we chose two time points that correspond to late infancy (4 weeks) and adulthood (12 weeks), a time frame in which several synaptic proteins are highly modulated (34). Although ASD can be reliably diagnosed by 3 years of age (35), ASD is a lifelong condition that deserves to be investigated with a dynamic outlook. It is hence fundamental to study ASD brains at several developmental time points, in order to optimize diagnostic and therapeutical interventions. Developmental changes of D1- and D2-family receptors have been demonstrated in the human brain (36). Recent PET imaging studies in humans with ASD investigated the D1 (37) and D2 binding (38) in DS. These two studies can only within limits be compared with our observations because (a) only adult subjects were considered; (b) no distinction between idiopathic and syndromic forms of ASD was made; (c) VS was not analyzed as an entity *per se*, even though it has already been considered in other human studies as potential therapeutical target in case of repetitive behavior (39, 40); and d) functional assays with a focus on differences in responses to tasks (38) or correlations to behavioral data (37) were performed.

The DA hypothesis of ASD is supported by alterations in the dopaminergic metabolism and transmission in several different mouse models such as *Df(h22q11)/+* mice (model for the human 22q11.2 microdeletion syndrome) (41), *Cntnap4* KO (KO model of a protein highly associated to ASD and schizophrenia) (42), *Ube3a* KO (model for Angelman syndrome) (43), *Chd8^+/−^* mice (model for the CHD8-related syndrome) (44), and VPA (model for the prenatal exposure to valproic acid) (45).

The models investigated in our study have also been analyzed by other groups regarding alterations in DA metabolism and neurotransmission. BTBR mice have no altered mRNA expression of D1 and D2 receptors in DS but reduced D2 postsynaptic signaling at adulthood (27). We also found no alterations in the receptor binding density for D1 in the DS, whereas D2 binding density was incremented (Figures 2A,B and 3A,B). Pharmacological interventions affecting DA signaling have proved effective in the BTBR line. The administration of p-cresol, a metabolite found elevated in children with ASD, leads to increased DA turnover in DS and VS of BTBR mice. Moreover, it aggravates repetitive behavior and social interaction deficits (46). Multi-targeting ligands such as ST-713 and ST-2223, both histamine H3- and DA D2-receptor antagonists, proved effective in reducing repetitive behavior and social deficits (47, 48). This evidence points again at an involvement of the dopaminergic systemic in BTBR mice, albeit the interplay with other neurotransmitter systems might complicate the general understanding. Moreover, it was recently shown that levels of tyrosine hydroxylase, the enzyme which catalyzes the first reaction in the biosynthesis of DA, are reduced in the DS and VTA but not in the VS of BTBR mice. Intranasal injection of DA could restore the levels of tyrosine hydroxylase and restore the observed social approach deficit. Interestingly, in the same study, *Fmr1* KO mice were also investigated. Contrary to the BTBR mice, no changes were found in tyrosine hydroxylase levels neither in the DS nor in the VS of *Fmr1* KO animals, despite an improvement of social preference upon administration of intranasal DA (25). This might seem to point at the fact, that different ASD models might have profoundly divergent molecular etiologies. In our study too, the receptor binding profiles to D1 receptor show no convergency in the BTBR and *Fmr1* models (Figures 2A,C). Nonetheless, at 12 weeks, we observed increased D2 receptor binding density in the VS of BTBR (Figure 3B), and *Shank3b* KO mice (Figure 3F). This is an interesting convergency concerning both an idiopathic and a genetic mouse model of ASD, which deserves to be more profoundly explored in future studies. A similar tendency was also observed in the *Fmr1* KO mice (Figure 3D).

Fragile X mental retardation protein, the RNA-binding protein encoded by the gene FMR1, has been proven as a crucial mediator of the integration between the D1- and D2-signaling and glutamatergic neurotransmission (49). In striatal cultures from *Fmr1* KO mice, reduced coupling of the D1 receptor with the Gs protein was detected upon stimulation with a D1 agonist. Moreover, an increased phosphorylation at serine sites was also registered (50). We also found alterations in the D1 binding density both in DS and VS at 4 but not 12 weeks in the *Fmr1* KO mice (Figures 2C,D). Such observations, however, pinpoint the fact that mapping the receptor binding density or the quantity of the receptor itself might not be enough to comprehend in depth the entity of the alterations. In a later study, DA release was measured in striatal slices at different time points. At 12 and 15 weeks, but not at 10 weeks, a significant decrease in the DA release was observed (51). In the *Fmr1* KO model hence, an increased D1 receptor phosphorylation is accompanied by a time-dependent fashion reduction of the DA release. A subsequent study confirmed reduced protein kinase A as a consequence of the reduced D1 signaling (52). Interestingly, protein kinase A is part of the cyclic adenosine monophosphate signaling, on which both D1 and D2 receptor families converge, either stimulating or inhibiting adenylyl cyclase (7). Furthermore, ultrastructural and electrophysiological alterations were found in the VS of *Fmr1* KO mice (53). We detected at 4 weeks a reduction of the D1 receptor binding both in DS and in VS. At 12 weeks of age, this was not observed (Figures 2C,D).

Although *Shank3* mutant mice are among the most deeply investigated monogenic models for ASD, studies regarding dopamine transmission remain unclear. This is even more surprising considering that the SHANK3 protein is enriched in the DS (54) and that available evidence in human patients with Phelan-McDermid syndrome points at major alterations in the DS (55, 56). Silencing SHANK3 in the VTA leads to reduced dopaminergic activity and impaired social preference, which can be reversed by modulation of glutamatergic transmission. Effects on the downstream targets of the VTA such as VS or DS were not investigated though (57). Deletion of SHANK3 in the DS using *Dlx5/6* and *Drd2-Cre* mice leads to increased perseverative explorative behavior. Moreover, deletion of SHANK3 in neurons expressing D1 or D2 leads to increased excitability in the DS (58). Altered glutamatergic transmission was however highlighted in the *Shank3b* model (59, 60). Although these two studies did not directly approach dopaminergic transmission, inhibition of protein kinase A was able to ameliorate the behavioral deficits of the *Shank3b* KO mice. Morphological and electrophysiological alterations of D2-expressing medium spiny neurons were also reported in 6–8 weeks old *Shank3b* KO mice (28). We also found increased binding to the D2 receptor both in DS and in VS at 12, but not 4 weeks of age (Figures 3E,F). In the same study, moreover, it was highlighted that voltage-gated calcium channels Cav1.3 are impaired in D2-expressing medium spiny neurons. As previously demonstrated (61, 62), SHANK3 mediates the regulation of D2 receptors on the Cav1.3 ones which, on turn, enable glutamatergic signaling. A recent *in vitro* study on brains from individuals with ASD also highlighted increased D2 mRNA in the caudate and putamen but unchanged D2 receptor binding (63). Interestingly, we found in the DS of *Fmr1* KO and *Shank3b* KO increased D2 binding density at adulthood (Figures 3D,F). The differences observed between that study and ours might be due to the different [^3^H] ligands used and to different analytical approaches. However, other mouse lines associated to ASD showed alterations in the D2 signaling. The mouse model of 16p11.2 deletion syndrome displays elevated number of D2 expressing neurons both in DS and VS (64). In the above-mentioned *Cntnap4* KO model, administration of haloperidol, a D2-antagonist, led to reduced repetitive behavior (42).

In conclusion, several lines of evidence show that the balance between D1 and D2 activation in the DS and VS is warranted (18). A disbalance toward higher activation of D1 signaling might lead, for example, to enhance stereotyped behavior (65, 66). Its modulation, on the opposite, either through optogenetics or pharmacologically, can reduce the repetitiveness (67–69). Trying to undermine the role of dopaminergic system in ASD is even more difficult considering that DA receptors D1 and D2 might form in DS and VS (and also in the hippocampus, cingulate cortex, and frontal cortex) heteromers with other G-coupled receptors such as metabotropic glutamate receptors, oxytocine-, serotonin-, and cannabinoid-receptors (70). Notably, both Aripiprazole and Risperidone, the only drugs approved so far from the FDA for ASD, act, even if with different mechanisms, pleiotropically on D2 and serotonin receptors. Both drugs were reported as effective against irritability and stereotypical behavior in idiopathic autism (71–73) and also proved effective in patients with Phelan-McDermid syndrome (74, 75) and Fragile-X syndrome (76, 77). As our results highlight, we found a significant increase of D2 receptor density in the VS at adulthood in the BTBR and *Shank3b* lines. A similar tendency was also observed in the *Fmr1* line. Our results hence appear to corroborate in a translational fashion, on the neuroanatomical level, evidence derived from pharmacological studies. Future experiments should be designed in order to tackle the intertwined molecular interplay between dopaminergic and other neurotransmitter systems. Targeted pharmacological intervention aiming at restoring the intracellular molecular cascades might prove effective, but further *in-vivo* investigations are needed.

## Data availability statement

The raw data supporting the conclusions of this article will be made available by the authors, without undue reservation.

## Ethics statement

Ethical review and approval was not required for the animal study because organ removal from mice for scientific purpose (the brain in this study) does not require approval by an ethics committee in Germany.

## Author contributions

SC, LN, PL, CJS, and MJS planned the autoradiographic experiments. SC, LN, and PL conducted the autoradiographic experiments and analyzed the data. SC and LN drafted the manuscript. PL, CJS, and MJS critically revised and edited the manuscript. All authors contributed to the article and approved the submitted version.

## Funding

LN was supported by an internal grant of the University Medical Center, Mainz (Stufe I), MJS by the German Research Foundation (DFG, Collaborative Research Center 1080, Project B10) and the Werner Reichenberger Foundation.

## Conflict of interest

The authors declare that the research was conducted in the absence of any commercial or financial relationships that could be construed as a potential conflict of interest.

## Publisher’s note

All claims expressed in this article are solely those of the authors and do not necessarily represent those of their affiliated organizations, or those of the publisher, the editors and the reviewers. Any product that may be evaluated in this article, or claim that may be made by its manufacturer, is not guaranteed or endorsed by the publisher.

## Abbreviations

[^3^H]: tritium
ASD: autism spectrum disorder
BTBR: black and tan brachyury
DA: dopamine
DS: dorsal striatum
FMR1: fragile X mental retardation 1
KO: knockout
SEM: standard error of the mean
SHANK: SH3 and multiple ankyrin repeat domains protein 3
VS: ventral striatum
VTA: ventral tegmental area
WT: wildtype
